# 

**Published:** 1874-08

**Authors:** 


ESTABLISHED IN 1855.

Volume XII. | AUGUST—1874. | Number 5.

### ATLANTA

### Medical and Surgical Journal.

MONTHLY: THREE DOLLARS PER ANNUM, IN ADVANCE.

ROBERT BATTEY, M.D.,

EDITOR.

WM. ABRAM LOVE, M.D., V. H. TALIAFERRO, M.D.,
ASSOCIATE EDITORS.

*PAX ET SCIENTIA, SED VERITAS SINE TIMORE.*

ATLANTA, GEORGIA:

*DUNLOP, WYNNE & CO., BOOK AND JOB PRINTERS.*

1874.

